# 174. Increase in *Candida auris* cases in New Jersey healthcare facilities during the COVID-19 pandemic — 2017–2020

**DOI:** 10.1093/ofid/ofab466.174

**Published:** 2021-12-04

**Authors:** Reed Magleby, Gabriel Innes, Diya Cherian, Jessica Arias, Jason Mehr, Kaitlin Forsberg, Meghan Lyman, Rebecca Greeley

**Affiliations:** 1 Centers for Disease Control and Prevention, PA; 2 New Jersey Department of Health, Trenton, New Jersey; 3 CDC, Atlanta, Georgia

## Abstract

**Background:**

*Candida auris* is a fungal pathogen associated with multidrug resistance, high mortality, and healthcare transmission. Since its U.S. emergence in 2017, to March 19, 2021, 1708 clinical infections were reported nationwide, of which 235 (13.8%) were reported in New Jersey. The New Jersey Department of Health (NJDOH) maintains *C. auris* surveillance in healthcare facilities (HCF) such as acute care hospitals, long-term acute care hospitals (LTACHs), and skilled nursing facilities, to monitor clinical infections and patient colonization. We aimed to characterize the epidemiology of *C. auris* infection and colonization among HCF patients during 2017–2020.

**Methods:**

HCFs report *C. auris* cases identified from clinical specimens and surveillance activities such as admission screenings and point prevalence surveys (PPS) to NJDOH. Cases are classified as either infection or colonization using National Notifiable Diseases Surveillance System case definitions. We analyzed cases reported during 2017–2020 to describe types of cases, facilities reporting cases, and demographics of affected patients. We analyzed PPS results to calculate percent positivity of tests from patients without previously identified infection and compared percent positivity between types of facilities. We examined quarterly trends for all variables before and after the COVID-19 pandemic peak in the second quarter of 2020.

**Results:**

During 2017–2020, 614 *C. auris* cases identified from clinical specimens were reported to NJDOH [243 (39.6%) infection, 371 (60.4%) colonization]; of these, 139 (57.2%) and 301 (81.1%) , respectively, were identified at long-term acute care hospitals (LTACHs). PPS percent positivity was higher at LTACHs (mean 7.6%) compared with all other facility types (mean 3.6%) for 13 of 16 quarters during 2017–2020. Case reports increased 2.6-fold from the Q2 2020 peak of the COVID-19 pandemic to Q3 2020.From Q1 to Q4 2020, PPS percent positivity increased from 4.8% to 10.5%.

Figure 1. *Candida auris* cases reported to New Jersey Department of Health, 2017–2020

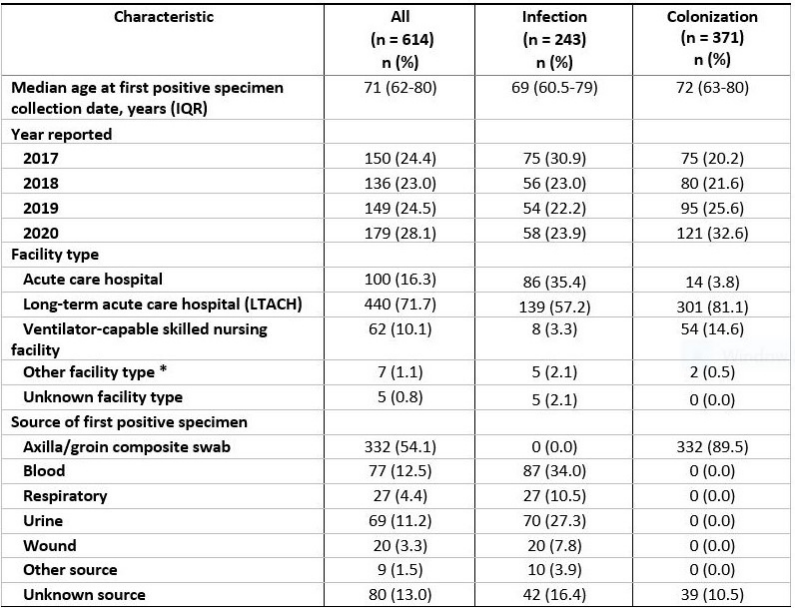

Figure 2. *Candida auris* test percent positivity among healthcare facility patients sampled for point prevalence surveys* and total number of C. auris point prevalence tests performed, New Jersey, 2017–2020. *Excluding individuals already known to be cases

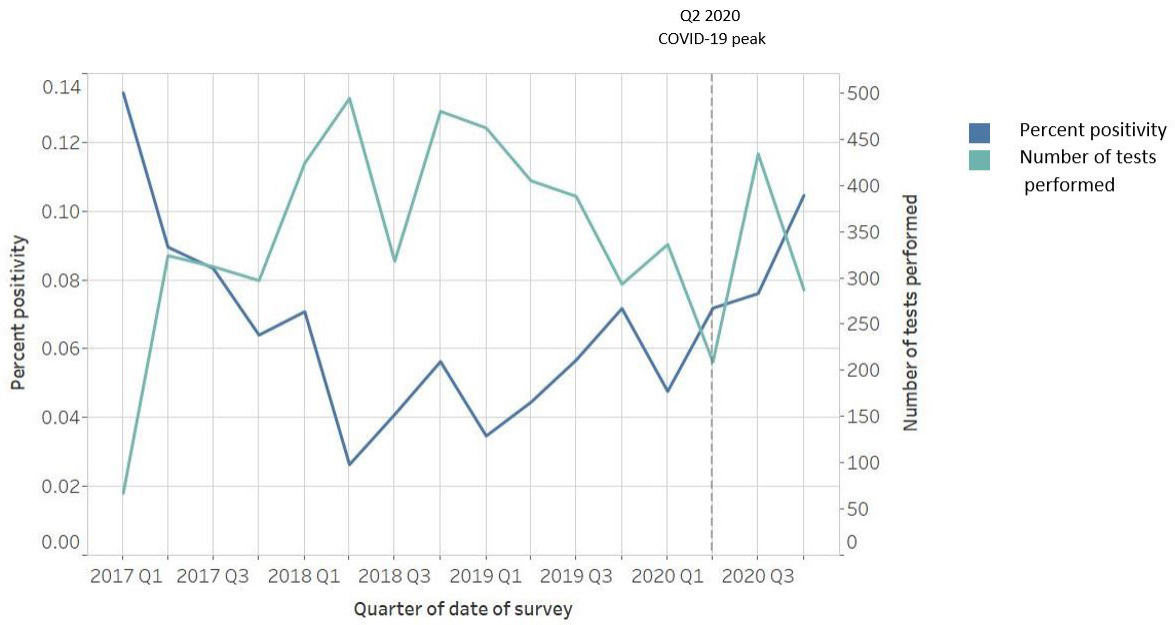

**Conclusion:**

The COVID-19 pandemic may have exacerbated *C. auris* transmission in HCF and potential causes should be further explored. LTACHs carry a disproportionate burden of patients colonized with *C. auris* and should be prioritized for surveillance and containment efforts.

**Disclosures:**

**All Authors**: No reported disclosures

